# Rethinking Vertigo Evaluation in the Emergency Department: Why Diagnosing Benign Paroxysmal Positional Vertigo (BPPV), Not With Early CT or MRI, Most Effectively Reduces Diagnostic Error

**DOI:** 10.7759/cureus.102040

**Published:** 2026-01-21

**Authors:** So Sakamoto

**Affiliations:** 1 Department of Emergency Medicine, Asahi General Hospital, Asahi, JPN

**Keywords:** bedside examination, benign paroxysmal positional vertigo, diagnostic error, emergency department, vertigo diagnosis

## Abstract

In emergency departments, early CT or MRI is frequently used in the evaluation of vertigo, largely driven by concern for posterior circulation stroke. However, early CT is insensitive to posterior fossa ischemia, and early MRI may be falsely negative in the first 24-48 hours. In contrast, benign paroxysmal positional vertigo (BPPV), the most common cause of acute vertigo, remains underdiagnosed despite its characteristic clinical features and effective bedside treatment. This editorial argues that the dominant diagnostic error in vertigo care is not failure to detect rare central causes, but failure to actively identify BPPV. By re-centering vertigo evaluation on careful history-taking, positional testing, and recognition of canal-specific nystagmus patterns, before reflexive imaging, clinicians can reduce diagnostic error and unnecessary testing while maintaining patient safety.

## Editorial

In many emergency departments across Japan, CT and MRI are obtained early in the evaluation of vertigo, sometimes even before a structured bedside examination is performed. This imaging-first approach reflects appropriate concern for missing a posterior circulation stroke, though this often drives imaging before structured bedside evaluation. Yet, early CT is insensitive to posterior fossa ischemia, and early MRI can be falsely negative in the first 24-48 hours. Meanwhile, the most common cause of acute vertigo--benign paroxysmal positional vertigo (BPPV)--remains underdiagnosed despite its high prevalence and clearly identifiable patterns. As Edlow and Newman-Toker note, the physical examination remains the most powerful tool for evaluating acute dizziness, yet it is frequently underutilized in emergency practice [1].

The dominant diagnostic error in vertigo care is not failing to detect rare, dangerous central causes--it fails to actively seek and confirm BPPV. Most cases involve the posterior semicircular canal, followed by horizontal canal variants. Posterior canal BPPV responds well to the Epley maneuver, whereas horizontal canal BPPV may present differently and require specific canalith repositioning techniques. Importantly, BPPV should not be conceptualized as a single entity diagnosed solely by the Dix-Hallpike test; rather, it is a spectrum of canal-specific disorders unified by their positional triggers and responsiveness to canalith repositioning maneuvers. Clinically, patients often describe their dizziness as “continuing” or “never going away,” yet careful history-taking reveals that each individual vertigo episode is brief and triggered by movement. Recognizing this pattern sharply lowers the pretest probability of central disease. In contrast, failure to pursue a bedside diagnosis maintains diagnostic uncertainty and drives unnecessary imaging.

The HINTS (Head Impulse, Nystagmus, Test of Skew) examination was originally reported as more sensitive than early MRI for detecting posterior circulation stroke, but only within the narrow population of acute vestibular syndrome (AVS). In real-world ED settings, especially in Japan, where older adults represent a large proportion of dizziness patients, HINTS is challenging to perform reliably. Weak or brief nystagmus, visual fixation, difficulty performing the head impulse test, and lack of Frenzel goggles all reduce diagnostic accuracy. Accurate application of HINTS requires appropriate training and experience, and misclassification of nystagmus or incomplete examination can undermine its diagnostic value in routine ED practice. Edlow and colleagues emphasize that HINTS is not a beginner’s test and should not be applied outside strict AVS criteria [2]. Misapplication risks generating false reassurance, which is more dangerous than diagnostic uncertainty.

When performed correctly, the Dix-Hallpike test produces characteristic positional symptoms and nystagmus that strongly suggest posterior canal BPPV, the most common subtype. BPPV, however, is fundamentally canal-specific: posterior, horizontal, and, less commonly, anterior canal variants each generate distinct nystagmus patterns and require appropriate repositioning maneuvers. The clinician’s goal is therefore not simply to identify “any BPPV,” but to determine whether the patient’s vertigo is positional and treatable with the correct canalith repositioning procedure.

Confirming BPPV effectively excludes central vertigo because each individual vertigo episode is brief, typically lasting only seconds, despite patients often perceiving their dizziness as continuous due to recurrent attacks. The symptoms are reproducibly triggered by specific positional changes, and characteristic positional nystagmus appears only when the appropriate provoking maneuver is performed. This nystagmus demonstrates a short latency after the positional change and a canal-specific direction, such as an upbeating torsional pattern in posterior canal involvement, and is typically absent at rest. Patients with BPPV also exhibit no focal neurological deficits, making a central cause highly unlikely. Identifying BPPV in this way eliminates the need for reflexive CT or MRI in most dizziness presentations. This does not imply that bedside findings alone are sufficient in all cases; rather, neuroimaging remains essential when bedside evaluation is incomplete, inconsistent, or reveals atypical features, regardless of patient age. As demonstrated by the validation of the STANDING algorithm, systematic positional testing and nystagmus evaluation outperform imaging in distinguishing benign from dangerous vertigo [3].

Posterior canal BPPV, the most common subtype, can be both diagnosed and treated at the bedside with canalith repositioning maneuvers, offering rapid symptom relief and preventing unnecessary diagnostic escalation. Even when BPPV is suspected, however, canalith repositioning maneuvers may fail if key prerequisites are overlooked. Common pitfalls include misidentifying the affected canal, insufficient head extension due to improper positioning or use of pillows, inadequate waiting time between positional steps, and insufficient explanation to the patient before the maneuver. These issues are not technical failures alone; they reflect incomplete assessment, rushed execution, and limited shared understanding between clinician and patient.

Recent evidence reinforces the primacy of bedside evaluation. Ohle and colleagues developed a clinical risk score for dangerous vertigo and demonstrated that the features most strongly associated with reduced stroke risk align with those consistent with BPPV [4]. Dangerous vertigo is far less common than benign causes, and bedside findings are more discriminative than imaging. CT cannot exclude a posterior circulation stroke. MRI, although superior, is limited early in the disease course. Overreliance on imaging risks false reassurance rather than diagnostic clarity.

Importantly, efforts to maximize sensitivity through exclusion-based strategies illustrate the trade-offs inherent in vertigo evaluation. A recent Japanese multicenter study proposed the EMERALD (Emergency Medicine, Registry Analysis, Learning and Diagnosis) Vertigo Rule to exclude central vertigo, achieving 100% sensitivity but only 20% specificity by incorporating symptoms such as vomiting and laboratory parameters, including blood glucose and lactate dehydrogenase [5]. While such approaches may reduce the risk of missed central pathology, they inevitably flag a large proportion of patients with benign peripheral vertigo, including most cases of BPPV, and often require blood testing and imaging. In the emergency department, this highlights the limitation of strategies that prioritize exclusion over diagnosis and reinforces the value of actively identifying BPPV at the bedside.

Emergency departments in Japan care for a disproportionately large number of older adults. Because stroke risk clearly increases with age, clinicians often default to imaging-first strategies in elderly patients out of caution. However, epidemiologic data consistently demonstrate that even among older adults, BPPV remains the most common cause of acute vertigo. The problem is not solely that stroke becomes more prevalent, but that clinicians tend to overestimate its probability while underrecognizing the age-related increase in benign positional vertigo. This mismatch between perceived and actual risk further illustrates why structured bedside assessment, rather than routine early imaging, should guide diagnostic reasoning, even in older populations.

Diagnostic practice in the ED is also shaped by system-level factors such as imaging availability, medico-legal concerns, and workflow pressures. These contextual influences help explain the persistence of imaging-first strategies and must be considered when promoting changes in practice. Effective vertigo evaluation, therefore, requires shifting from reflexive imaging toward a more deliberate, structured approach to bedside reasoning. Trainees should be explicitly taught that each vertigo episode in BPPV is brief, typically lasting seconds, even if patients perceive symptoms as “continuous” due to frequent recurrences; that positional changes reproducibly provoke symptoms; that spontaneous nystagmus at rest is usually absent, with characteristic positional nystagmus appearing only when appropriate provoking maneuvers are performed; and that canal-specific positional testing, including the Dix-Hallpike and appropriate horizontal canal tests, s indispensable for both diagnosis and guiding canalith repositioning. When supervising physicians explain not just what they order but why, diagnostic thinking becomes visible and teachable. Transparency helps dismantle outdated beliefs and cultivates diagnostic stewardship-an essential skill in CT-rich environments like Japan, where imaging availability can accelerate both effective and ineffective habits.

Emergency medicine exists at the intersection of urgency, uncertainty, and diagnostic pressure. Within this environment, an important and often underrecognized threat is failing to recognize common benign causes, even as clinicians appropriately remain vigilant for rare central etiologies. Confident, consistent diagnosis of BPPV is the most effective strategy to reduce imaging overuse, prevent diagnostic delay, and improve care for dizzy patients. Rather than opposing imaging, a tiered risk assessment that integrates structured bedside evaluation with judicious neuroimaging offers a more balanced and safer approach to vertigo care in the emergency department. By restoring bedside skills to the center of vertigo evaluation, we train future clinicians to think critically rather than reflexively and foster an emergency care culture rooted in evidence rather than habit.
